# Editorial: Culture and emotion in educational dynamics, volume III

**DOI:** 10.3389/fpsyg.2025.1662995

**Published:** 2025-10-03

**Authors:** Silvia Cristina da Costa Dutra, Enrique H. Riquelme, Dario Paez

**Affiliations:** ^1^Departamento de Psicología y Sociología, Universidad de Zaragoza, Zaragoza, Spain; ^2^Departamento de Diversidad y Educación Intercultural, Universidad Catolica de Temuco, Temuco, Chile; ^3^Programa Doctorado en Educación y Ciencias Sociales, Temuco Catholic University, Temuco, Chile; ^4^Universidad Andres Bello, Santiago, Chile

**Keywords:** culture, emotion, educational, dynamic, methodology in educational research

This volume continues an editorial line that seeks to integrate the understanding of emotions in educational processes with a culturally situated approach. Previous volumes laid the theoretical foundations of this interrelationship, while this new compendium delves into its practical and methodological expressions in diverse contexts. Thus, the reader is invited to explore a critical mapping of emotions as a constitutive part of educational work, from the perspective of those who teach, learn, support, and transform.

Volume III of “*Culture and emotion in educational dynamics*” expands the analysis of the complex interrelationship between cultural and emotional factors that shape contemporary educational contexts. This editorial offers a thematic review of the multidimensional content that constitutes this ever-evolving field of study.

The current work is structured around four thematic axes: the first examines emotional intelligence and its assessment methods in different educational communities; the second focuses on teacher wellbeing and professional trajectory, considering aspects such as identity construction, institutional support, and emotions related to professional practice; the third block explores emotional dynamics and psychosocial factors involved in language learning and intercultural student experiences; finally, the fourth axis addresses family participation and intercultural sensitivity in educational spaces.

The first thematic axis of the volume focuses on emotional intelligence as a key competence that permeates the educational experience. The research gathered here shows how the development of this skill can be promoted through pedagogical approaches that are contextualized and culturally relevant.

Block 1: Emotional Intelligence in Diverse Educational Communities Emotional intelligence (EI) is recognized as a crucial factor for wellbeing and success in the educational sphere. Its development can be fostered through specific pedagogical approaches, as investigated by the study of Lasekan et al. This work explores the role of emotional vocabulary in enhancing EI among future teachers of English as a foreign language (EFL), using the Headway series. Its objective was to quantify, categorize, and develop a systematic teaching model to improve EI. Through a mixed-methods approach, the study revealed a progressive increase in emotional vocabulary as competence levels advanced. Positive emotions were more frequent at beginner levels, while negative and neutral ones increased at higher stages, supporting the development of self-regulation and empathy. The proposed model offers a structured framework that addresses key components of EI such as self-awareness, social skills, and emotional regulation, representing a valuable contribution to teacher training programs.

In the second block, the focus shifts to teachers, analyzing how their identity trajectories, working conditions, and emotional wellbeing affect educational quality. The studies presented here advocate for a comprehensive view of teacher professional development.

Block 2: Teacher Wellbeing and Professional Development: Identity, Support, and Emotions Teacher professional identity and educator wellbeing are crucial for an effective educational system. The study by Luo et al. explores how different perceived school goal structures affect the professional identity of kindergarten teachers in China, considering the mediating role of the satisfaction of basic psychological needs (SBPN) and the moderating role of a growth mindset. With a sample of 1,475 teachers, the results showed that learning and performance goal structures indirectly affected professional identity through SBPN, although only the learning goal structure had a direct effect. Interestingly, the growth mindset only moderated the relationship between performance goal structure and SBPN. This study provides a theoretical basis and practical guidance for improving teacher professional identity in the Chinese sociocultural context.

Emotional support provided by teachers also plays a fundamental role in student performance. Wu and Yu investigate the influence of formative assessment on academic achievement, exploring the mediating role of teachers' emotional support. Analyzing data from 280 secondary students in southern China, they confirmed that teachers' emotional support mediates the relationship between formative assessment and academic performance, highlighting the importance of integrating emotional support strategies in formative assessment.

Professional honor is another important factor for teachers, especially in rural areas. Chen et al. examine the influence of demographic factors (gender, age, and region) on the professional honor of 1,320 rural teachers in China. Their analyses revealed that female teachers reported higher levels of professional honor, teachers in the Eastern Region showed more honor than those in the Western Region, and those aged 31 to 40 exhibited the lowest professional honor. These findings are key to addressing challenges in the prestige and retention of rural teachers.

Finally, professional burnout is a significant concern. The study by Liang and Yin analyzes burnout in university counselors in China and the moderating role of emotional intelligence in the relationship between emotional labor and burnout. A survey of 520 counselors indicated that burnout increases with age, being highest between ages 26 and 30. Addressing burnout from both individual and organizational dimensions is recommended, with a focus on improving emotional intelligence.

The third axis offers a deep look at the emotions that emerge in linguistic and intercultural learning contexts. The research highlights how factors such as teacher support, anxiety, enjoyment, and perseverance intertwine to shape students' experiences.

Block 3: Emotional and Cultural Dynamics in Language Learning and Intercultural Student Experience Learning a foreign language is a process deeply influenced by emotional factors and perceived support. The study by Pan et al. investigates the associations between students' grit and perceived teacher support with their performance in the Chinese language, considering the mediating role of emotions (enjoyment, anxiety, and boredom) in Thai secondary classrooms. With 665 students, the results indicated that teacher support, grit, and emotions were significantly associated with performance. While all three emotions mediated the relationship between teacher support and performance, only enjoyment mediated the association between perseverance and performance. The study highlights the importance of fostering perseverance, supportive environments, and addressing students' emotional needs.

Understanding the theoretical foundations of emotions in this context is essential. Wu and Kabilan provide a review of theories on emotion in the language classroom, focusing on their conceptualization and causality. They argue that few studies have deeply investigated underlying theoretical frameworks and identify the effects of interactions among cognitive, psychological, social, and contextual factors in emotional development.

Emotional interactions in the classroom are dynamic. The study by Jiang explores emotional contagion between adult English students and their teacher in China. It identified negative emotions (student anxiety provoking teacher self-doubt) and positive emotions (student calm promoting teacher tranquility). These findings challenge traditional assumptions by identifying students as primary initiators of emotional contagion.

Vocal emotion recognition also has a cultural dimension. Cheng et al. investigated cross-regional cultural recognition of emotion in adolescents' voices. Their experiments confirmed that Chinese adolescents demonstrated a greater ability to recognize vocal emotions within their own cultural group, a skill that is accentuated with greater cultural differences.

Social support is vital for students in new environments. The study by Fu validated the Index of Sojourner Social Support (ISSS) among internal migrant university students in China, exploring how support facilitates psychological adjustment. With 1,692 students, it was found that instrumental support, in particular, significantly influenced students' psychological adjustment to the host culture. The study concludes that the scale is psychometrically sound and that instrumental support is key to adjustment.

The final block focuses on the role of families and the importance of fostering intercultural sensitivity in schools. It examines the conditions that favor or hinder the participation of migrant families in school processes, as well as the impact of institutional climate on such participation.

Block 4: Family Participation and Intercultural Sensitivity in Educational Settings Family-school collaboration is fundamental, especially in immigration contexts. The study by Bilbao et al. examines the role of intercultural sensitivity in predicting immigrant parents' participation in early childhood education in Chile. With a sample of 347 parents, it was shown that greater intercultural sensitivity predicted higher participation. Parents with lower intercultural sensitivity were 75% more likely to report “almost never” participating. Among the barriers, parents who perceived that the school lacked a special approach for immigrant families were 3.79 times more likely to report low participation. These findings highlight the importance of promoting intercultural sensitivity in school communities.

Expanding on this line, Mera-Lemp et al. investigate the relationships between school cultural diversity climate, cultural sensitivity, and school participation among 751 Venezuelan and Peruvian immigrant parents in Chile. The results showed that sociodemographic variables had limited effects on participation, while the cultural diversity climate and cultural sensitivity had greater influence. This study emphasizes the importance of schools' approach to cultural diversity and parents' intercultural competencies in their commitment to their children's education.

The studies compiled in this volume provide empirical evidence and robust conceptual frameworks to understand how emotions and culture intertwine in school life. Beyond specific contexts, the investigations engage in dialogue to propose a more human, inclusive, and situated educational vision. In doing so, this volume aims to contribute to an education that recognizes emotional and cultural diversity as an asset, rather than a barrier, thereby strengthening teaching and learning processes at all levels of the educational system.

